# Do microbes play a role in Alzheimer's disease?

**DOI:** 10.1111/1751-7915.14462

**Published:** 2024-04-09

**Authors:** Zoë A. P. Williams, Leonie Lang, Sarah Nicolas, Gerard Clarke, John Cryan, David Vauzour, Yvonne M. Nolan

**Affiliations:** ^1^ Department of Anatomy and Neuroscience University College Cork Cork Ireland; ^2^ APC Microbiome Ireland University College Cork Cork Ireland; ^3^ Norwich Medical School, Faculty of Medicine and Health Sciences University of East Anglia Norwich UK; ^4^ Department of Psychiatry and Neurobehavioural Science University College Cork Cork Ireland

## Abstract

Alzheimer's disease is a complex and progressive condition that affects essential neurological functions such as memory and reasoning. In the brain, neuronal loss, synaptic dysfunction, proteinopathy, neurofibrillary tangles, and neuroinflammation are the hallmarks of Alzheimer's disease pathophysiology. In addition, recent evidence has highlighted that microbes, whether commensal or pathogenic, also have the ability to interact with their host and to regulate its immune system, therefore participating in the exchanges that lead to peripheral inflammation and neuropathology. Because of this intimate relationship, bacteria, viruses, fungi, and protozoa have been implicated in the development of Alzheimer's disease. Here, we bring together current and most recent evidence of the role of microbes in Alzheimer's disease, raising burning questions that need to be addressed to guide therapeutic approaches and potential prophylactic strategies.

## Graphical abstract

Is there a role of microbes in Alzheimer's disease progression? What remains to be addressed to guide therapeutic approaches and potential prophylactic strategies to delay Alzheimer's disease onset.

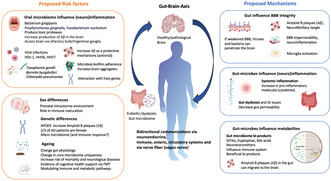

## INTRODUCTION

With increasing longevity and declining fertility our global population is becoming disproportionality aged. Ageing is associated with an increased incidence of Alzheimer's disease (AD), which is now recognised as one of the most significant health challenges globally with important socio‐economic dimensions (Alzheimer's disease facts and figures, 2022). AD is characterised by a progressive decline in cognition and memory, as well as emotional and personality changes (Knopman, Amieva, et al., 2021). The pathological hallmarks include extracellular amyloid beta (Aβ) plaques (parenchymal Aβ1‐42 peptides and vascular amyloid deposits with shorter Aβ1‐40 peptides), intraneuronal accumulation of neurofibrillary tangles, and neuroinflammation, primarily in the cortex and hippocampus, which spread to other brain regions as the disease progresses (Braak & Braak, 1991; DeTure & Dickson, 2019; Thal et al., 2002). Interestingly, recent research from animal models of AD and post‐mortem human tissue points to the presence of beta‐amyloid in the gut, with the ratio of Aβ1‐42 to Aβ1‐40 being higher in the gut than in the brain of human patients (Jin et al., 2023).

The amyloid cascade hypothesis posits that AD pathogenesis is caused by the accumulation of Aβ in the brain, triggering a cascade of inflammation, tau accumulation and the generation of neurofibrillary tangles, synaptic dysfunction, and subsequent neuronal death (Hardy & Allsop, 1991; Mattsson‐Carlgren et al., 2021; Therriault et al., 2021). However, after over 400 clinical trials targeting brain‐derived Aβ, such a strategy has not proven to be very effective (Banik et al., 2015; Cummings et al., 2014; Geldenhuys & Darvesh, 2015; Knopman, Jones, & Greicius, 2021). Furthermore, recent developments in amyloid‐targeting monoclonal antibody medications only offer marginal benefits for cognitive decline (van Dyck et al., 2023). Plasma levels of tau are reported as reliable biomarkers for AD pathology (Ashton et al., 2021; Palmqvist et al., 2020) with predictive capacity for future AD diagnosis (Lantero Rodriguez et al., 2020), and tau targeted treatments have also been explored, but with limited efficacy (Florian et al., 2023; Teng et al., 2022). Multiple recent genome wide association studies report a central role of the innate immune system in AD pathophysiology (Bellenguez et al., 2022; Kunkle et al., 2019; Wightman et al., 2021), pointing to the multifactorial nature of the disease. The complexity of AD resides in the fact that genes and the immune system do not work in isolation but are manipulated by other players such as peripheral and environmental factors. The gut microbiota is particularly receptive to environmental influences and the microbiota‐gut‐brain axis (MGBA) is emerging as a key target for investigation in AD. Recent research on the involvement of microbes in age‐related cognitive decline and AD is bolstered by findings from over a decade ago proposing Aβ as an antimicrobial peptide (Connell et al., 2022; Cryan et al., 2020; Soscia et al., 2010). Thus, there is growing support from research observations for a microbial hypothesis of AD, and it is tempting to speculate that it could have significant implications for how researchers approach the development of treatments (Table S1).

## AMYLOID BETA: A VILLAIN OR HERO?

Several lines of evidence support the role of Aβ as an antimicrobial peptide, which defends the brain against invading pathogens. Aβ has a similar structure to other antimicrobial peptides and acts by triggering an innate immune response to pathogen entry into the brain (Eimer et al., 2018). Specifically, Aβ variants can trigger agglutination of microbes by binding to surface carbohydrates found on microbes, which prevents microbial entry into host cells and tissues and/or promotes phagocytosis by microglia (Eimer et al., 2018; Spitzer et al., 2016; Vojtechova et al., 2022). Increased levels of microbes in the brains of AD patients (Alonso, Pisa, Marina, et al., 2014; Dominy et al., 2019; Jamieson et al., 1991; Pisa, Alonso, Rábano, et al., 2015) have been linked to increased antimicrobial activity (Soscia et al., 2010) and might also reflect on a weaker blood–brain barrier (BBB) integrity of this population (Vigasova et al., 2021). Moreover, elevated Aβ protected against infection with the bacteria *Salmonella* Typhimurium in a mouse model of AD (Kumar et al., 2016). However, ageing, or genetic vulnerabilities can have negative consequences on an individual's Aβ system. For example, the apolipoprotein E4 (*APOE4*) gene variant is the stronger genetic risk factor for AD, driving earlier and more abundant Aβ deposition in the brain (Corder et al., 1993; Kloske & Wilcock, 2020; Saunders et al., 1993). Prolonged elevation of Aβ triggers over activation of microglia, increasing neuroinflammation and ultimately leading to neurodegeneration and the progression of AD symptoms (Moir et al., 2018). In AD patients, microglia appear to be dysfunctional and their overactivation/reduced phagocytosing capacity could explain the accumulation of Aβ in AD (Cameron & Landreth, 2010). However, while it is plausible that increased deposition of Aβ, and consequent neuroinflammation and AD progression occurs in response to the presence of microbes in the brain, neuroinflammation may also arise from systemic infections (perhaps due to a weakened immune system) (Knox et al., 2022) or microbial dysbiosis (Thapa et al., 2023). The infectious disease hypothesis posits that a pathogen may be the cause of AD (Mawanda & Wallace, 2013; Seaks & Wilcock, 2020), particularly in late onset AD (Sochocka et al., 2017). This pathogen can act directly or indirectly, by triggering an inflammatory response (Li et al., 2021) (Table S1).

## ALZHEIMER'S DISEASE AND VIRAL INFECTION

A role for the virus herpes simplex type 1 (HSV1) – the cause of cold sores – in AD was first theorised in 1982 when its presence was reported in human trigeminal ganglia. The finding that brain regions affected in AD are the same brain regions damaged by HSV encephalitis contributed to speculation that latent viruses may travel via trigeminal ganglia to the brain, become reactivated, and cause AD neurodegeneration (Ball, 1982). This theory was supported in 1991 when Professor Ruth Itzhaki discovered increased levels of herpes virus in *postmortem* brain samples of patients with AD (Jamieson et al., 1991). Since then, it has been reported that HSV‐1 infection can seed Aβ in both in vitro and in vivo experiments (Eimer et al., 2018; Wozniak et al., 2007). More recently, evidence suggests that HSV1 preferentially targets the hippocampus, a brain region particularly impacted in AD (reviewed by Yong et al., 2021). Additionally, HSV1 infection in patients who carry the *APOE4* gene variant increases the risk of developing AD (Linard et al., 2020). HSV1 may access the brain in early‐ or mid‐life via an olfactory route to the olfactory bulb, through vertical transmission maternally during pregnancy, or may be dormant in trigeminal ganglia (reviewed by Duarte et al., 2019). Reactivation of HSV1 in later life, due to stressors such as other infections, head trauma, or an ageing immune system, contributes to chronic neuroinflammation and consequent neurodegeneration. This is supported by a study that found recurrent reactivation of HSV1 by thermal stress in mice which led to the accumulation of Aβ and tau in the cortex and/or hippocampus with consequent cognitive deficits (Chiara et al., 2019). Interestingly, Aβ was reported to prevent the entry of HSV1 into infected MRC‐5 cells in vitro thereby inhibiting their replication (Bourgade et al., 2015).

The proposal of a relationship between herpes virus and AD was greatly strengthened by a study that found herpes viruses HHV6 and HHV7 in post‐mortem brain tissue of patients with AD in three independent cohorts (Readhead et al., 2018). Moreover, it was shown that viruses may interact with host genes that are risk factors for AD, such as genes regulating the processing of APP (Readhead et al., 2018). In fact, some viruses such as HSV1, Epstein–Barr virus (EBV), and Vaccinia virus (VACV) have all been reported to feature co‐expression networks specific to virus‐host systems (Aguirre & Guantes, 2023; López‐Lastra, 2022). As a result, several host protein interactors are differentially expressed during viral infection (Karunakaran et al., 2022). Of major interest, it has recently been observed that APP interacts with SARS‐CoV‐2 transmembrane fusion proteins, known as spike proteins, which not only facilitates viral entry but also worsens the progression of Aβ‐associated disease (Chen, Chen, et al., 2023).

**FIGURE 1:**
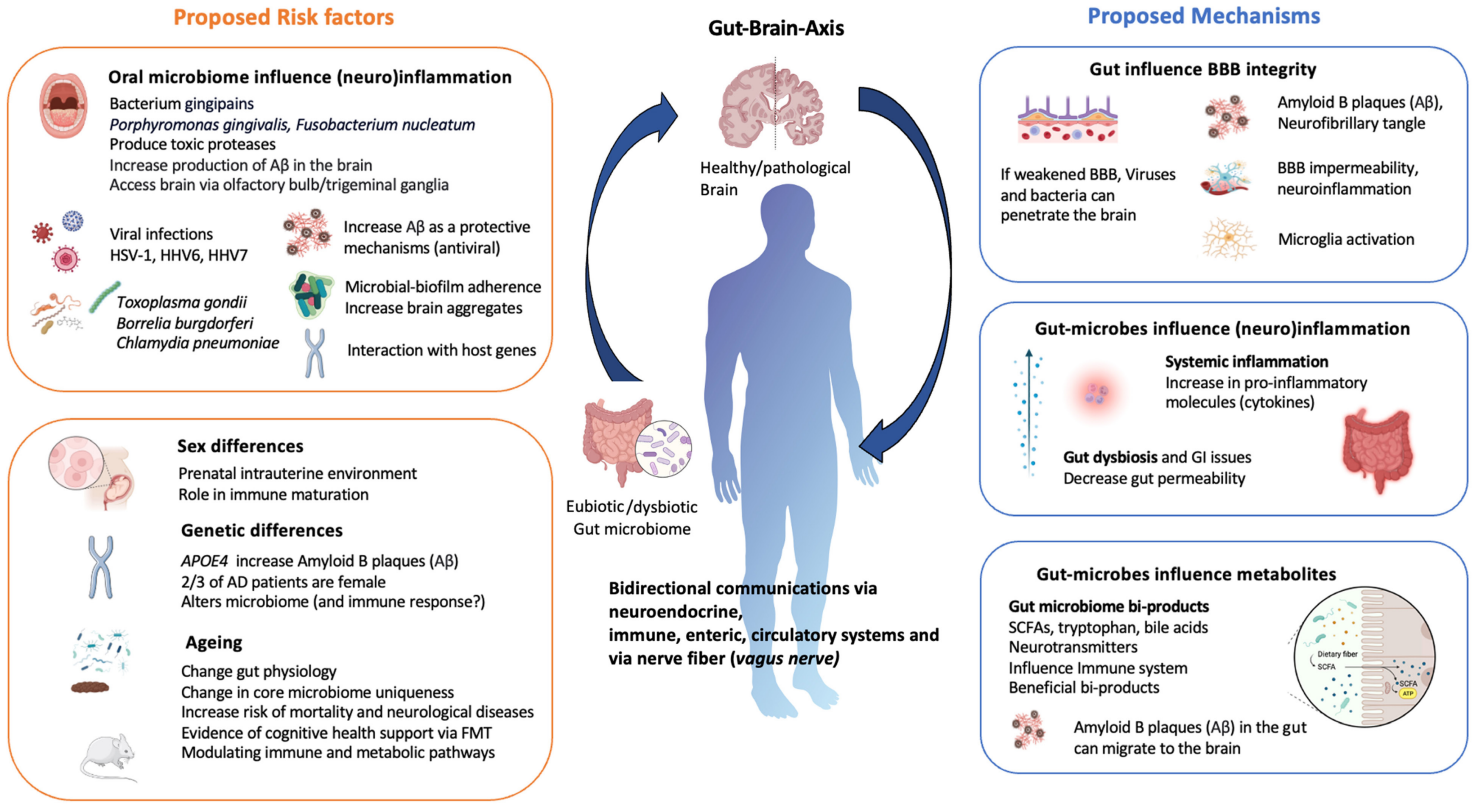
Proposed mechanisms and risk factors involved in brain health.

A seminal retrospective cohort study from Taiwan has shown that patients with HSV infections treated with an antiherpetic had decreased the risk of developing dementia by 90% (Tzeng et al., 2018). Vaccinations against other viruses (herpes zoster and/or tetanus, diphtheria, and pertussis) have also been associated with decreased risk of dementia (Wiemken et al., 2022), and causal evidence of the herpes zoster vaccination against dementia risk has been reported (Eyting et al., 2023). However, a clinical trial in patients with early‐stage AD found only a slight and highly variable improvement in mini mental state examination scores and no change in cerebrospinal fluid (CSF) levels of tau after 4 weeks of treatment with the antiviral valacyclovir (Weidung et al., 2022). Thus, although there is increasing support for a viral role in AD more research is needed to turn this knowledge into the development of efficacious treatments. It should be noted that although HSV1 is a major focus, over 20 types of viral infection have recently been associated with an increased risk of dementia (Levine et al., 2023) (Figure 1 and Table S2).

## ALZHEIMER'S DISEASE AND FUNGAL AND PROTOZOAN INFECTION

Various fungal species and fungal infections have been reported in the brains of AD patients (Alonso, Pisa, Marina, et al., 2014; Pisa, Alonso, Rábano, et al., 2015; Salama et al., 2018), and the protozoan parasite *Toxoplasma gondii* has been implicated in AD pathogenesis. A recent meta‐analysis exploring the connection between *T. gondii* and AD reported a small effect size, but findings were based on a small number of studies and thus the authors could not infer a conclusive result (Nayeri Chegeni et al., 2019). The potential role of fungi in AD pathology is further supported by the detection of fungal DNA and proteins in AD patients, specifically, *postmortem* brain tissues (Pisa, Alonso, Juarranz, et al., 2015), blood serum, and CSF (Alonso, Pisa, Rábano, & Carrasco, 2014). However, the role of fungi and protozoa has been overshadowed by exploration into other microbes, and thus more research is needed to understand the role of fungal infection and protozoan infection in AD (Figure 1 and Table S2).

## ALZHEIMER'S DISEASE AND BACTERIAL INFECTION

Bacteria have been implicated as triggers in AD pathogenesis, particularly *Porphyromonas gingivalis*, the periodontal bacteria that cause gum disease (Beydoun et al., 2020; Dominy et al., 2019; Sparks Stein et al., 2012). *P. gingivalis* infection in mice is reported to increase Aβ in peripheral macrophages (Nie et al., 2019) and has been found in *postmortem* brain samples from AD patients (Dominy et al., 2019). Oral bacteria in the brains of individuals with AD have been reported to be circa seven‐fold higher when compared to cognitively healthy individuals (Miklossy, 2008). However, whether microbes have simply gained pathogenic entry into the brain, or are forming an ecosystem is up for debate, as the notion of microbes within the brain is not without controversy. Although some question whether microbes have an intimate relationship with the brain, forming a sort of brain microbiome, other cautions against drawing conclusions from low biomass samples as results may indicate contamination rather than local habitation (Kennedy et al., 2023).

The mechanism underlying the effects of *P. gingivalis* in AD is proposed to be via the production of toxic proteases called gingipains, as levels of gingipains in the brain correlate with tau pathology in AD patients. Moreover, gingipain inhibition was reported to have neuroprotective effects after *P. gingivalis* infection in mice, reducing the levels of Aβ and markers of neuroinflammation (Dominy et al., 2019). However, a *P. gingivalis* targeting drug failed to display significant benefits in clinical trials and concerns of liver toxicity halted the clinical trials (Atuzaginstat | ALZFORUM).

Other bacterial species have also been hypothesised to play a role in AD. The DNA sequence of *Chlamydia pneumoniae*, a common respiratory pathogen in elderly people, was reported in the brain tissue of 80% of patients with AD (Gérard et al., 2006), and a more recent study in mice showed that *Chlamydia pneumoniae* could infect the central nervous system, potentially travelling via the olfactory and trigeminal nerves (Chacko et al., 2022). *Borrelia burgdorferi*, which is associated with Lyme disease (Miklossy, 2011), has also been hypothesised to play a role in AD pathogenesis as it has been found to colocalise with plaques in AD patients, and its in vitro infection of mammalian cells induced an increase in the levels of Aβ and phosphorylated tau (Senejani et al., 2022). Furthermore, a common pathogen involved in the development of periodontitis, *Fusobacterium nucleatum*, has been reported to play a role in AD. Antibodies against *F. nucleatum* were detected in the serum of cognitively impaired patients as well as in the serum of AD patients (Wu et al., 2022). Importantly, the causal relationship was confirmed with evidence of an exacerbation of cognitive symptoms, Aβ accumulation, and tau phosphorylation in 5XFAD mice topically infected with *F. nucleatum* to induce periodontitis (Wu et al., 2022) (Figure 1 and Table S2).

## ALZHEIMER'S DISEASE AND THE GUT MICROBIOME

Changes in gastrointestinal physiology during ageing lead to alterations in the intestinal microbiota and may in turn influence the brain (Boehme et al., 2023). Microbes are part of the complex ecosystem of microorganisms called the microbiome and have co‐evolved with humans and play commensal, symbiotic, and pathogenic roles in their human host. The microbiome is localised in body sites such as skin, mucosa, or gut, which is the largest component of the human microbiome. Crucial findings connecting the gut microbiome health with brain health ushered forth the research field which has been known as the microbiota‐gut‐brain axis (Bercik et al., 2011; Bravo et al., 2011; Heijtz et al., 2011; Neufeld et al., 2011). This bidirectional link between the gut and the brain comprises neural (via the *vagus nerve*), immune, metabolic, and endocrine pathways (Cryan et al., 2019). The initial study showing a disrupted gut microbiome in AD patients (Vogt et al., 2017) has been supported by studies showing that Alzheimer's patients display alterations in their gut microbiota composition, and in particular a decrease in microbial diversity. Microbial diversity is a potential hallmark of healthy ageing, and ageing leads to divergences in the gut microbiome composition (Claesson et al., 2011). Gut microbial characteristics in AD have shown that the relative abundance of the bacterial phylum *Firmicutes*, which produces beneficial metabolites, is lower and the abundance of *Bacteroidetes* which have inflammatory activity is higher in AD patients (Chen, Zhou, et al., 2023; Saji et al., 2019). Of interest, increased *Bacteroidetes* and/or low microbial uniqueness was recently linked to higher risk of mortality in a 4‐year follow‐up study, with distinct microbial metabolic outputs in the blood (phenylalanine/tyrosine metabolites) (Wilmanski et al., 2021). In this regard, AD patients are reported to have an increased prevalence of pro‐inflammatory genera in the gut microbiota, which positively correlates with pro‐inflammatory cytokine expression in the blood (Cattaneo et al., 2017). Moreover, cognitive performance in AD has been negatively associated with the inflammatory pathobiont genera *Desulfovibrio* (Grabrucker et al., 2023). Changes in the gut microbiome composition have recently been reported to precede the early stages of disease pathology (Sheng et al., 2022), suggesting a role of the microbiome in AD development, which could be qualified as a potential biomarker and an early signal of the disease. Recent studies have reported a genetic overlap between AD and gastrointestinal disorders in humans (Adewuyi et al., 2022) and correlations between the gut microbiome genera and AD associated genes (Cammann et al., 2023), indicating an interaction between the gut microbiome and genetics in AD pathogenesis. For example, it is well documented in mammals and humans that *APOE* genotype alters the gut microbiome diversity and metabolism (Maldonado Weng et al., 2019; Tran et al., 2019; Zajac et al., 2022).

Preclinical studies using animal models of AD with the depleted gut microbiota, derived by crossing with germ‐free mice (genetically and environmentally devoid of microorganisms) or by using either long‐term broad‐spectrum combinatorial antibiotic treatment, have demonstrated that the lack of gut microbiota induces a reduction of Aβ pathology, a delay in memory‐deficits, and an altered microglial activation status, which occurs in concert with a reduction in tau accumulation (Harach et al., 2017; Mezö et al., 2020; Minter et al., 2016; Seo et al., 2023). It has also been shown that transplantation of the gut microbiota from an AD mouse model can transfer the AD‐related memory impairment to wild‐type mice (Kim et al., 2021). These studies provided the first indication of a relationship between the gut microbes and AD, at least in animal models and point to microglial‐mediated Aβ clearance mechanisms. A recent study found that transferring the gut microbiota from Alzheimer's patients to healthy young rats also transferred the cognitive impairments (Grabrucker et al., 2023). This research has confirmed a causal role of the gut microbiota in AD and highlights that an understanding of microbes in brain–body interaction may lead to novel approaches for AD (Figure 1 and Table S2).

## BURNING QUESTIONS

### Are microbes a main player or a supporting act in Alzheimer's pathology?

Are microbes the cause of AD or are they simply a reflection of homeostatic dysregulation seen in the disease by acting as opportunistic pathogens that infect a weakened host. Are the microbes localised in the brain due to the weakened BBB, or could this weakened BBB be arising from alterations in the gut microbiota that exacerbate disease pathology via influencing the immune system and barrier permeability? Longitudinal clinical studies which can determine when microbes enter the brain and/or when alterations in the gut microbiome occur in relation to the AD onset will be crucial for ascertaining the microbe‐AD relationship (Itzhaki et al., 2020). This is pertinent because AD pathology is reported to begin 20 years before AD symptomatology appears, making it not only difficult to establish a causal link but also difficult to determine an optimal time to initiate treatment (Golde et al., 2018). By assessing the gut microbiota biomarkers at presymptomatic stages or earlier in the life course such as in middle age (Ances et al., 2023; Ferreiro et al., 2023), it is plausible that the lack of gut homeostasis and related changes in metabolites and neuroactive compounds could be identified as a prodromal symptom of AD. Studies such as these could also reveal whether the microbes, their metabolites, or both, are potentially responsible for AD symptoms and disease progression.

### How one crisis aggravates another: What is the link between COVID‐19 and the risk of developing AD?

New research highlights that SARS‐CoV‐2 infection can cause atrophy in brain regions related to AD (Douaud et al., 2022). Moreover, SARS‐CoV‐2 patients display tau hyperphosphorylation implicated in AD (Reiken et al., 2022), and an increased risk of AD following SARS‐CoV‐2 infection has been suggested (Wang et al., 2022). Given the high infection rate by SARS‐CoV‐2 globally, parsing apart the relationship between microbes and AD is becoming increasingly important. This knowledge will be critical for guiding not only the development of treatment strategies for AD but also prevention strategies such as the use of antivirals.

### How does biological sex influence the relationship between microbes and Alzheimer's disease?

As researchers work to close the biological sex gap, increasing evidence highlights sex specific differences in AD. A greater number of AD patients are female (Rajan et al., 2021). Females have a different gut microbiome composition from males (Shobeiri et al., 2022), and sex differences in immune cells and their responsivity have been reported in patients with AD. Specifically, peripheral blood leukocytes display a lower response to viral infection ex vivo in female compared to male AD patients (Coales et al., 2022; Sochocka et al., 2022). A study exploring gene expression in the brain and blood of patients with AD suggested AD immune dysregulation may be a specific feature to females (Paranjpe et al., 2021), and a preclinical study reported sex specific responsivity of microglia to antibiotic treatment in a mouse model of AD (Dodiya et al., 2019). It was also suggested that the higher prevalence of AD in females might be due to the modulation of oestrogen levels (Wharton et al., 2009), which, interestingly, was observed in relation to changes in the microbiome during adolescence and menopause (Peters et al., 2022), and there is now an increasing awareness that menopause may be a critical stage for AD treatment (Scheyer et al., 2018). Taken together, it is likely that microbial action in AD has sex specific features. Thus, understanding the relationship between biological sex, AD, and microbes will be necessary for the development of adequate and equitable treatment and prevention. Speaking of minority groups, it is essential that longitudinal studies exploring a causal relationship between microbes, metabolites, and AD are ethically and racially diverse (Raman et al., 2021), as some studies report an almost two times increased risk of dementia in African Americans (Barnes, 2022).

### Could precision medicine increase the efficacy of microbe targeted treatments?

With increasing research reporting a role of different microbes in AD pathology, finding a universally effective treatment is currently challenging; thus, a precision medicine‐based approach may be more effective. However, with the sheer quantity of microbes, the lack of targeted treatments, and the developmental trajectory of AD highlighting that symptom appearance occurs years after disease initiation, the complexity of timing for prevention and treatment is challenging. The findings from FMT studies open a promising avenue for microbial mediated attenuation of the negative cognitive effects of Alzheimer's disease, which could be part of precision medicine approach. A recent study employed a precision medicine‐based approach that utilised antimicrobial, lifestyle, and hormonal screening and treatment resulting in 84% of AD patients displaying improvements (Toups et al., 2022). In this regard, it could be considered that the best defence against AD may be a good offence. Instead of waiting for AD to arise along with the dysregulation of the immune system and microbiome that comes with age, the ageing global population could be equipped with immuno‐ and microbial‐ modulatory lifestyle habits to face the coming storm of neurodegeneration.

### The gut microbiome and AD

The intestinal microbiome plays a key role in maintaining host health, including the brain. However, before designing microbiome targeted therapies, we need to understand the ecosystem of microbes, microbial metabolites, and microbe host interactions throughout the lifespan, as recently proposed by Ratsika et al. (2023). Clarifying our understanding of what makes a “healthy/unhealthy” gut microbiome is paramount, as there is currently no consensus and proper definition of “gut dysbiosis” (Brüssow, 2020). Until now, the GMBA field has focused on bacteria, but expanding our exploration to all microbes and microbe‐microbe interactions will be crucial for understanding and targeting this “second brain” when our “first brain” fails.

## CONCLUSION

The field of AD research has long focused on Aβ as the main player in disease pathogenesis, but with research now highlighting the potential involvement of microbes in AD pathology; coupled with evidence of an antimicrobial role of Aβ, microbes may soon take centre stage. However, the role of microbes in AD is not in opposition to other theories but is rather unifying. By providing an explanation for the increased presence of Aβ and inflammation in AD pathology, microbes bring together the Aβ hypothesis, the antimicrobial protection hypothesis, and the infectious disease hypothesis. The aetiology of AD is complex and most likely cannot be connected to one specific mechanism. Indeed, from the gut to oral microbiome, viruses, fungi, and protozoa microbes are proposed as causal in AD. Extending the research in AD to encompass the role of microbes, provides novel opportunities for treatment development and hope for the future.

## AUTHOR CONTRIBUTIONS

**Zoë A. P. Williams:** Conceptualization; writing – original draft; writing – review and editing. **Leonie Lang:** Conceptualization; writing – original draft; writing – review and editing. **Sarah Nicolas:** Conceptualization; writing – original draft; writing – review and editing. **Gerard Clarke:** Conceptualization; writing – review and editing. **John Cryan:** Conceptualization; writing – review and editing. **David Vauzour:** Conceptualization; writing – review and editing. **Yvonne M. Nolan:** Conceptualization; writing – review and editing.

## FUNDING INFORMATION

No funding information provided.

## CONFLICT OF INTEREST STATEMENT

The authors declare no conflict of interest.

## Supporting information


Data S1.

## Data Availability

Data sharing not applicable to this article as no datasets were generated or analysed during the current study.
