# Supplementary material for: Do microbes play a role in Alzheimer's disease?
Source: Microb Biotechnol. 2024 Apr 9;17(4):e14462. doi: 10.1111/1751-7915.14462 (PMC11003713; doi:10.1111/1751-7915.14462)
Supplement: Supplementary file 1 — Data S1. [file MBT2-17-e14462-s001.docx]

**Table S1.** Evidence of infections related to AD phenotype

| **Pathological AD characteristics** | **Results and evidence** | **References** |
| --- | --- | --- |
| **Rodent studies** | | |
| Amyloid beta | Inhibition of gingipain exerts neuroprotective effects and decrease levels of amyloid beta and markers of neuroinflammation | (Dominy *et al.*, 2019) |
| **Human studies** | | |
| Amyloid beta | Genetic variations (amyloid beta) are linked to microbial entry and induction/promotion of phagocytosis | (Spitzer *et al.*, 2016; Eimer *et al.*, 2018; Vojtechova *et al.*, 2022) |
|  | Connective mutations *APP* gene observed in AD patients. Only a subset of AD patients, suggesting other triggers | (Corder *et al.*, 1993; Saunders *et al.*, 1993) |
| Tau pathology | Biochemical analysis of AD patients revealed increased plasma levels of tau, can be used as predictive biomarkers | (Palmqvist *et al.*, 2020; Ashton *et al.*, 2021) |
| Amyloid beta and tau | Synergistic relationship between amyloid beta and tau as amyloid beta modulates tau tangle deposition, cognition depends on levels of tau and amyloid | (Mattsson‐Carlgren *et al.*, 2021; Therriault *et al.*, 2021) |
| General AD pathology | Numerous genome-wide association studies (GWAs) have highlighted tau and amyloid pathways, and the role of the innate immune system in AD. | (Kunkle *et al.*, 2019; Wightman *et al.*, 2021; Bellenguez *et al.*, 2022) |
|  | Evidence of temporal lobe homogenates analysis of AD patients revealed an increased antimicrobial activity, with negative correlation between amyloid beta and *C. albicans* growth | (Soscia *et al.*, 2010) |
|  | Evidence from seminal retrospective cohort study in AD patients revealed that antiherpetic treatment decreased by 90% the risk of developing dementia in patients with HSV infections | (Tzeng *et al.*, 2018) |
|  | Results from human virus vaccination (zoster, tetanus, diphtheria, pertussis) revealed that the combination of vaccinations reduced the risk of AD, with timid cognitive improvements. No change in CSF tau and neurofilament levels (valacyclovir treatment for 4 weeks) | (Weidung *et al.*, 2022; Wiemken *et al.*, 2022; Eyting *et al.*, 2023) |
| **Human and rodent studies** | | |
| Amyloid beta | Evidence from Human and animal post-mortem tissues found higher amyloid beta presence in the gut than in the brain with higher Amyloid beta 42/amyloid beta 40 ratio | (Jin *et al.*, 2023) |

**Table S2.** Evidence of pathogens involved in the hallmarks of AD

| **Study model** | **Species** | **Causes and effects** | **References** |
| --- | --- | --- | --- |
| **Rodent studies** | | | |
| **Virus** | | | |
| Upper-lip abrasion infection with HSV-1 2uL suspension in rodents | Herpes simplex type 1 (HSV1) | Environmental stress (e.g thermal stress) leads to reactivation of HSV1 and to amyloid beta and tau within the cortex and hippocampus, associated with cognitive deficits | (Chiara *et al.*, 2019) |
| **Bacteria** | | | |
| 5XFAD mice Infected topically | *Fusobacterium nucleatum* | Exacerbation of cognitive symptoms, beta-amyloid accumulation, and tau phosphorylation | (Wu *et al.*, 2022) |
| Mouse model of Alzheimer’s disease | *Salmonella* Typhimurium | Increased amyloid beta antimicrobial peptide after infection, increased survival | (Kumar *et al.*, 2016) |
| **Human studies** | | | |
| **Fungus** | | | |
| Blood serum and CSF of AD patients | NC | Fungal proteins and DNA detected in blood serum and CSF | (Alonso *et al.*, 2014) |
| Post-mortem human brain tissue with AD | Fungal protein *Tubulin* | Presence of tubulin in brain tissues | (Salama *et al.*, 2018) |
| **Protozoan parasite** | | | |
| Post-mortem human brain tissue with AD | *Taxoplasma gondii* | Positive correlation with AD, based on small effect size studies | (Nayeri Chegeni *et al.*, 2019) |
| **Virus** | | | |
| Post-mortem human brain tissue with AD | Herpes simplex type 1 (HSV1) | HSV1 preferentially targets the hippocampus. Increased levels of herpes virus in | (Jamieson *et al.*, 1991; Yong *et al.*, 2021) |
| *In vitro,* MRC-5 cells | Herpes simplex type 1 (HSV1) | Amyloid beta peptides decreased amount of viral DNA up to 50% in MRC-5 cells infected by HSV1, amyloid beta inhibits viral replication | (Bourgade *et al.*, 2015) |
| Post-mortem human brain tissue with AD | Herpes HHV6 and HHV7 | Viruses interaction with host genes APP | (Readhead *et al.*, 2018) |
|  | SARS-CoV-2 | Atrophy of brain regions in AD patients and tau hyperphosphorylation | (Douaud *et al.*, 2022; Reiken *et al.*, 2022) |
|  | Herpes simplex type 1 (HSV1) | Traveling through the trigeminal ganglia, migrate into the brain by anterograde transport, causing neurodegenerative diseases and memory and learning disorders reminiscent of AD | (Ball, 1982) |
| **Bacteria** | | | |
| *In vitro* AD model | *Borrelia burgdorferi* | Increased amyloid beta and phosphorylated tau | (Senejani *et al.*, 2022) |
| Post-mortem human brain tissue with AD | *Porphyromonas gingivalis* | Periodontal bacteria causing gum disease detected in post-mortem brain tissues, producing toxic proteases gingipain | (Dominy *et al.*, 2019) |
|  | *Chlamydia pneumoniae* | DNA reported in the brain of 80% of AD patients, infect CNS via olfactory and trigeminal nerves | (Gérard *et al.*, 2006; Chacko *et al.*, 2022) |
| Serum of AD and cognitively impaired patients | *Fusobacterium nucleatum* | Antibodies detected in the serum of cognitively impaired and AD patients | (Wu *et al.*, 2022) |
| Human AD patients | *Firmicutes/Bacteroidetes* | Lower levels of *Firmicutes* and higher *Bacteroidetes.* Higher ratio of Firmicutes/Bacteroidetes (F/B) ratio in demented AD patients. Conflicting results in human AD patients with both decreased/increased levels. Overall alterations of gut microbiome | (Vogt *et al.*, 2017; Saji *et al.*, 2019) |

**References**

Alonso, R., Pisa, D., Marina, A.I., Morato, E., Rábano, A., and Carrasco, L. (2014) Fungal infection in patients with Alzheimer’s disease. *J Alzheimers Dis* **41**: 301–311.

Ashton, N.J., Pascoal, T.A., Karikari, T.K., Benedet, A.L., Lantero-Rodriguez, J., Brinkmalm, G., et al. (2021) Plasma p-tau231: a new biomarker for incipient Alzheimer’s disease pathology. *Acta Neuropathol* **141**: 709–724.

Ball, M.J. (1982) Limbic predilection in Alzheimer dementia: is reactivated herpesvirus involved? *Can J Neurol Sci* **9**: 303–306.

Bellenguez, C., Küçükali, F., Jansen, I.E., Kleineidam, L., Moreno-Grau, S., Amin, N., et al. (2022) New insights into the genetic etiology of Alzheimer’s disease and related dementias. *Nat Genet* **54**: 412–436.

Bourgade, K., Garneau, H., Giroux, G., Le Page, A.Y., Bocti, C., Dupuis, G., et al. (2015) β-Amyloid peptides display protective activity against the human Alzheimer’s disease-associated herpes simplex virus-1. *Biogerontology* **16**: 85–98.

Chacko, A., Delbaz, A., Walkden, H., Basu, S., Armitage, C.W., Eindorf, T., et al. (2022) Chlamydia pneumoniae can infect the central nervous system via the olfactory and trigeminal nerves and contributes to Alzheimer’s disease risk. *Sci Rep* **12**: 2759.

Chiara, G.D., Piacentini, R., Fabiani, M., Mastrodonato, A., Marcocci, M.E., Limongi, D., et al. (2019) Recurrent herpes simplex virus-1 infection induces hallmarks of neurodegeneration and cognitive deficits in mice. *PLOS Pathogens* **15**: e1007617.

Corder, E.H., Saunders, A.M., Strittmatter, W.J., Schmechel, D.E., Gaskell, P.C., Small, G.W., et al. (1993) Gene Dose of Apolipoprotein E Type 4 Allele and the Risk of Alzheimer’s Disease in Late Onset Families. *Science* **261**: 921–923.

Dominy, S.S., Lynch, C., Ermini, F., Benedyk, M., Marczyk, A., Konradi, A., et al. (2019) Porphyromonas gingivalis in Alzheimer’s disease brains: Evidence for disease causation and treatment with small-molecule inhibitors. *Sci Adv* **5**: eaau3333.

Douaud, G., Lee, S., Alfaro-Almagro, F., Arthofer, C., Wang, C., McCarthy, P., et al. (2022) SARS-CoV-2 is associated with changes in brain structure in UK Biobank. *Nature* **604**: 697–707.

Eimer, W.A., Kumar, D.K.V., Shanmugam, N.K.N., Rodriguez, A.S., Mitchell, T., Washicosky, K.J., et al. (2018) Alzheimer’s Disease-Associated β-Amyloid Is Rapidly Seeded by Herpesviridae to Protect against Brain Infection. *Neuron* **99**: 56-63.e3.

Eyting, M., Xie, M., Heß, S., and Geldsetzer, P. (2023) Causal evidence that herpes zoster vaccination prevents a proportion of dementia cases. 2023.05.23.23290253.

Gérard, H.C., Dreses-Werringloer, U., Wildt, K.S., Deka, S., Oszust, C., Balin, B.J., et al. (2006) Chlamydophila (Chlamydia) pneumoniae in the Alzheimer’s brain. *FEMS Immunology & Medical Microbiology* **48**: 355–366.

Jamieson, G.A., Maitland, N.J., Wilcock, G.K., Craske, J., and Itzhaki, R.F. (1991) Latent herpes simplex virus type 1 in normal and Alzheimer’s disease brains. *J Med Virol* **33**: 224–227.

Jin, J., Xu, Z., Zhang, L., Zhang, C., Zhao, X., Mao, Y., et al. (2023) Gut-derived β-amyloid: Likely a centerpiece of the gut–brain axis contributing to Alzheimer’s pathogenesis. *Gut Microbes* **15**: 2167172.

Kumar, D.K.V., Choi, S.H., Washicosky, K.J., Eimer, W.A., Tucker, S., Ghofrani, J., et al. (2016) Amyloid-β peptide protects against microbial infection in mouse and worm models of Alzheimer’s disease. *Sci Transl Med* **8**: 340ra72.

Kunkle, B.W., Grenier-Boley, B., Sims, R., Bis, J.C., Damotte, V., Naj, A.C., et al. (2019) Genetic meta-analysis of diagnosed Alzheimer’s disease identifies new risk loci and implicates Aβ, tau, immunity and lipid processing. *Nat Genet* **51**: 414–430.

Mattsson‐Carlgren, N., Janelidze, S., Bateman, R.J., Smith, R., Stomrud, E., Serrano, G.E., et al. (2021) Soluble P‐tau217 reflects amyloid and tau pathology and mediates the association of amyloid with tau. *EMBO Mol Med* **13**: e14022.

Nayeri Chegeni, T., Sarvi, S., Moosazadeh, M., Sharif, M., Aghayan, S.A., Amouei, A., et al. (2019) Is Toxoplasma gondii a potential risk factor for Alzheimer’s disease? A systematic review and meta-analysis. *Microbial Pathogenesis* **137**: 103751.

Palmqvist, S., Janelidze, S., Quiroz, Y.T., Zetterberg, H., Lopera, F., Stomrud, E., et al. (2020) Discriminative Accuracy of Plasma Phospho-tau217 for Alzheimer Disease vs Other Neurodegenerative Disorders. *JAMA* **324**: 772–781.

Readhead, B., Haure-Mirande, J.-V., Funk, C.C., Richards, M.A., Shannon, P., Haroutunian, V., et al. (2018) Multiscale Analysis of Independent Alzheimer’s Cohorts Finds Disruption of Molecular, Genetic, and Clinical Networks by Human Herpesvirus. *Neuron* **99**: 64-82.e7.

Reiken, S., Sittenfeld, L., Dridi, H., Liu, Y., Liu, X., and Marks, A.R. (2022) Alzheimer’s-like signaling in brains of COVID-19 patients. *Alzheimer’s & Dementia* **18**: 955–965.

Saji, N., Murotani, K., Hisada, T., Tsuduki, T., Sugimoto, T., Kimura, A., et al. (2019) The relationship between the gut microbiome and mild cognitive impairment in patients without dementia: a cross-sectional study conducted in Japan. *Sci Rep* **9**: 19227.

Salama, M., Shalash, A., Magdy, A., Makar, M., Roushdy, T., Elbalkimy, M., et al. (2018) Tubulin and Tau: Possible targets for diagnosis of Parkinson’s and Alzheimer’s diseases. *PLoS ONE* **13**: e0196436.

Saunders, A.M., Roses, A.D., Pericak-Vance, M.A., Dole, K.C., Strittmatter, W.J., Schmechel, D.E., et al. (1993) Apolipoprotein E &isin;4 allele distributions in late-onset Alzheimer’s disease and in other amyloid-forming diseases. *The Lancet* **342**: 710–711.

Senejani, A.G., Maghsoudlou, J., El-Zohiry, D., Gaur, G., Wawrzeniak, K., Caravaglia, C., et al. (2022) Borrelia burgdorferi Co-Localizing with Amyloid Markers in Alzheimer’s Disease Brain Tissues. *J Alzheimers Dis* **85**: 889–903.

Soscia, S.J., Kirby, J.E., Washicosky, K.J., Tucker, S.M., Ingelsson, M., Hyman, B., et al. (2010) The Alzheimer’s disease-associated amyloid beta-protein is an antimicrobial peptide. *PLoS One* **5**: e9505.

Spitzer, P., Condic, M., Herrmann, M., Oberstein, T.J., Scharin-Mehlmann, M., Gilbert, D.F., et al. (2016) Amyloidogenic amyloid-β-peptide variants induce microbial agglutination and exert antimicrobial activity. *Sci Rep* **6**: 32228.

Therriault, J., Pascoal, T.A., Sefranek, M., Mathotaarachchi, S., Benedet, A.L., Chamoun, M., et al. (2021) Amyloid‐dependent and amyloid‐independent effects of Tau in individuals without dementia. *Ann Clin Transl Neurol* **8**: 2083–2092.

Tzeng, N.-S., Chung, C.-H., Lin, F.-H., Chiang, C.-P., Yeh, C.-B., Huang, S.-Y., et al. (2018) Anti-herpetic Medications and Reduced Risk of Dementia in Patients with Herpes Simplex Virus Infections-a Nationwide, Population-Based Cohort Study in Taiwan. *Neurotherapeutics* **15**: 417–429.

Vogt, N.M., Kerby, R.L., Dill-McFarland, K.A., Harding, S.J., Merluzzi, A.P., Johnson, S.C., et al. (2017) Gut microbiome alterations in Alzheimer’s disease. *Sci Rep* **7**: 13537.

Vojtechova, I., Machacek, T., Kristofikova, Z., Stuchlik, A., and Petrasek, T. (2022) Infectious origin of Alzheimer’s disease: Amyloid beta as a component of brain antimicrobial immunity. *PLOS Pathogens* **18**: e1010929.

Weidung, B., Hemmingsson, E.-S., Olsson, J., Sundström, T., Blennow, K., Zetterberg, H., et al. (2022) VALZ-Pilot: High-dose valacyclovir treatment in patients with early-stage Alzheimer’s disease. *Alzheimers Dement (N Y)* **8**: e12264.

Wiemken, T.L., Salas, J., Morley, J.E., Hoft, D.F., Jacobs, C., and Scherrer, J.F. (2022) Comparison of rates of dementia among older adult recipients of two, one, or no vaccinations. *Journal of the American Geriatrics Society* **70**: 1157–1168.

Wightman, D.P., Jansen, I.E., Savage, J.E., Shadrin, A.A., Bahrami, S., Holland, D., et al. (2021) A genome-wide association study with 1,126,563 individuals identifies new risk loci for Alzheimer’s disease. *Nat Genet* **53**: 1276–1282.

Wu, H., Qiu, W., Zhu, X., Li, X., Xie, Z., Carreras, I., et al. (2022) The Periodontal Pathogen Fusobacterium nucleatum Exacerbates Alzheimer’s Pathogenesis via Specific Pathways. *Front Aging Neurosci* **14**: 912709.

Yong, S.J., Yong, M.H., Teoh, S.L., Soga, T., Parhar, I., Chew, J., and Lim, W.L. (2021) The Hippocampal Vulnerability to Herpes Simplex Virus Type I Infection: Relevance to Alzheimer’s Disease and Memory Impairment. *Frontiers in Cellular Neuroscience* **15**:.
